# The difficult symbolic construction of physicians’ and nurses’ experiences working in COVID-19 intensive care units: A qualitative study on reports at a university public hospital in Southeastern Brazil

**DOI:** 10.1192/j.eurpsy.2022.1321

**Published:** 2022-09-01

**Authors:** E. Turato, F. Silva, L. Guerra, J. Cavalcante, A.P. Gasparotto, R. Aoki

**Affiliations:** State University of Campinas, Laboratory Of Clinical-qualitative Research, Campinas, Brazil

**Keywords:** Qualitative research, psychological meanings, Covid-19, health professionals

## Abstract

**Introduction:**

Humanistic studies that explore symbolic aspects of the experience of working on the COVID-19 frontline are necessary. Do these professionals have psychic time to symbolize such acute experiences? We expect these preliminary findings of this research provide subsidies for discussing psychological management in groups with these professionals.

**Objectives:**

To interpret emotional meanings reported by physicians and nurses on their experiences of working at COVID-19 intensive care units.

**Methods:**

Clinical-qualitative design. Data collection with semi-directed interviews with open-ended questions in-depth applied to a sample of six professionals, closed by theoretical information saturation, in a Brazilian university general hospital. Trigger question: “Talk about psychological meanings of your experience in face of management of patients with COVID-19 at ICU”. Data treatment by the Seven Steps of the Clinical-Qualitative Content Analysis. Theoretical framework of Medical Psychology using Balintian concepts.

**Results:**

We raised 3 categories. (1) Psychic time and absence of symbolization in face of the pandemic; (2) Denial as a defense or psychosocial adaptation mechanisms; (3) Tensions and family support: triggers of ambivalent emotional experiences.

**Conclusions:**

Raw experience of COVID-19 pandemic did not allow for realization of symbolization. Psychological defenses are manifested, either to maintain balance or to deny the existence of dangers related to mental health. Presence of families and health team confirm that the feeling of loneliness is avoided. Anxieties related to the fear of contamination are recurrent. There is dual relationship regarding the emotional experiences of health professionals, but the data point to importance of looking at how these individuals perceive and experience the pandemic.

**Disclosure:**

No significant relationships.

